# Influenza viral vectors expressing the *Brucella* OMP16 or L7/L12 proteins as vaccines against *B. abortus* infection

**DOI:** 10.1186/1743-422X-11-69

**Published:** 2014-04-10

**Authors:** Kaissar Tabynov, Abylai Sansyzbay, Zhailaubay Kydyrbayev, Bolat Yespembetov, Sholpan Ryskeldinova, Nadezhda Zinina, Nurika Assanzhanova, Kulaisan Sultankulova, Nurlan Sandybayev, Berik Khairullin, Irina Kuznetsova, Boris Ferko, Andrej Egorov

**Affiliations:** 1 The Research Institute for Biological Safety Problems, Zhambulskaya oblast, Kordaiskiy rayon, Gvardeisky, Republic of Kazakhstan; 2 HSC Development GmbH, Tulln, Austria

**Keywords:** Vaccine, Influenza virus, Vector, OMP16, L7/L12, *B. abortus* infection

## Abstract

**Background:**

We generated novel, effective candidate vaccine against *Brucella abortus* based on recombinant influenza viruses expressing the *Brucella* ribosomal protein L7/L12 or outer membrane protein (Omp)-16 from the NS1 open reading frame. The main purpose of this work was to evaluate the safety, immunogenicity and protectiveness of vaccine candidate in laboratory animals.

**Methods and Results:**

Four recombinant influenza A viral constructs of the subtypes Н5N1 or H1N1 expressing the *Brucella* proteins L7/L12 or Omp16 were obtained by a reverse genetics method: Flu-NS1-124-L7/L12-H5N1, Flu-NS1-124-Omp16-H5N1, Flu-NS1-124-L7/L12-H1N1 and Flu-NS1-124-Omp16-H1N1. Despite of substantial modification of NS1 gene, all constructs replicated well and were retain their *Brucella* inserts over five passages in embryonated chicken eggs (CE). Administration of the mono- or bivalent vaccine formulation via prime-boost intranasal (*i.n*.), conjunctival (*c*.) or subcutaneous (*s.c.*) immunization was safe in mice; no deaths, body weight loss or pathomorphological changes were observed over 56 days. Moreover, guinea pigs vaccinated *i.n.* with vaccine vectors did not shed the vaccine viruses through their upper respiratory tract after the prime and booster vaccination. These findings confirmed the replication-deficient phenotype of viral vectors. The highest antibody response to *Brucella* antigen was obtained with constructs expressing L7/L12 (ELISA, GMT 242.5-735.0); whereas the highest T-cell immune response- with construct expressing Omp16 (ELISPOT, 337 ± 52-651 ± 45 spots/4×10^5^cells), which was comparable (*P > 0.05*) to the response induced by the commercial vaccine *B. abortus 19*. Interestingly, *c.* immunization appeared to be optimal for eliciting T-cell immune response. In guinea pigs, the highest protective efficacy after challenge with *B. abortus* 544 was achieved with Omp16 expressing constructs in both monovalent or bivalent vaccine formulations; protective efficacy was comparable to those induced by a commercial live *B. abortus* 19 vaccine.

**Conclusion:**

Thus, influenza vectors expressing *Brucella* protective antigens can be developed as novel influenza vectored vaccine against *B. abortus* infection.

## Background

*Brucella abortus* is a facultative intracellular pathogen capable of infecting and causing disease in both domestic animals and humans [1]. At present, brucellosis among cattle is prevented using live attenuated vaccines from the strains *B. abortus* 19 or RB51. These vaccines possess a high immunogenic effectiveness, but have a number of serious disadvantages, primarily related to their ability to induce abortion in pregnant cows, secretion of the vaccine strain into the milk of vaccinated animals when they are used in adult cattle and the difficulty of differentiating between vaccinated animals and infected animals (only a concern for the *B. abortus* 19) [2]. Furthermore, both strains can cause systemic brucellosis in humans [3].

Given that *B. abortus* is an intracellular pathogen, the main criterion for new candidate vaccines is their ability to elicit a cellular immune response in animals. It is well recognized that the two key components of the protective reaction in infected animals are the formation of Th1 CD4^+^ lymphocytes secreting interferon-gamma (IFN-γ), a critical cytokine which is required to regulate the anti-brucellosis activity of macrophages [4], and CD8^+^ T lymphocytes that lyse *Brucella*-infected cells [5].

Attempts by various research groups to elicit effective Th1 CD4^+^ and CD8^+^ T cell anti-brucellosis immune responses have resulted in the development of subunit (recombinant protein) vaccines [6-14] and DNA vaccines [15-20], however in terms of protective efficacy, subunit and DNA vaccines are still inferior to commercial live attenuated vaccines.

An alternative strategy for the development of safe and effective brucellosis vaccines is the use of live genetically-modified vectors, i.e., non-pathogenic microorganisms (bacteria and viruses) that express a *Brucella* antigen. To date, *Escherichia coli*[21], *Salmonella enterica*[22], *Ochrobactrum anthropi*[23] and *Semliki Forest virus* (SFV) [24] have been used as vectors for expressing *Brucella* proteins *in vivo*. It has been shown that the tested bacterial (intracellular) and viral vectors are capable of infecting a wide range of cell types and expressing *Brucella* antigens within the infected cells. Furthermore, in all cases, Th1 CD4^+^ and CD8^+^ T-cell anti-brucellosis immune responses were elicited in immunized animals [21-24].

In view of the positive results obtained using live viral vectors and the practical advantages of the reverse genetics method, which enables genetic manipulation of RNA-containing viruses [25,26], we propose that recombinant influenza A viruses expressing the *Brucella* L7/L12 or Omp16 proteins may potentially represent a novel candidate vector vaccine against brucellosis. According to published data, L7/L12 ribosomal protein and Omp16 are immunodominant *B. abortus* proteins that elicit a cellular immune response (Th1 and CD8^+^ T cells) [8-10,13,14,16,20,22]. The influenza A virus contains a segmented genome consisting of eight negative-strand RNA fragments. Of these, the smallest fragment (NS), encoding two proteins: viral nonstructural protein (NS1) and nuclear export protein (Nep), is convenient target for genetic manipulation since NS1 is able to tolerate foreign sequences exceeding its own length [27]. Thus, the ORF of NS1 was used for inserting *Brucella* sequences in this study. The А/Puerto Rico/8/34 (H1N1) strain was used as the backbone for obtaining influenza A virus vectors expressing *Brucella* L7/L12 or Omp16 sequences in a form of fusion proteins with N- terminal 124 amino acid residues of NS1.

The mouse is the animal model most extensively used to study chronic infection caused by *Brucella* spp. [28]. Therefore, there are several reports of experimental work employing other laboratory animals are susceptible to experimental infection with *Brucella* spp. Guinea pigs are probably the most susceptible laboratory animal species to *Brucella* infection. Guinea pigs inoculated subcutaneously with infectious doses of *B. abortus* develop a persistent bacteremia for 6 weeks after infection, whereas the attenuated *B. abortus* S19 is cleared from the blood at one week after infection [29]. Therefore, the guinea pig model may be considered valuable for the evaluation of candidate vaccine strains [11]. All classic *Brucella* species were pathogenic for guinea pigs [28]. Accordingly, as the animal model for evaluating the protectiveness of our vaccine candidates we used guinea pigs.

In this work, we demonstrate that our novel recombinant Influenza Viruses expressing the *Brucella* proteins L7/L12 or Omp16, and combinations of thereof (bivalent vaccine formulation) elicited a T-cell immune response in mice after a prime-boost immunization regime via various immunization routes, and also provided guinea pigs with a high degree of protection against the virulent strain *B. abortus* 544, which was comparable to that offered by a commercial live vaccine produced from *B. abortus* 19.

## Results

### Recombinant influenza A viruses expressing B. abortus proteins L7/L12 or Omp16

**Figure 1:**
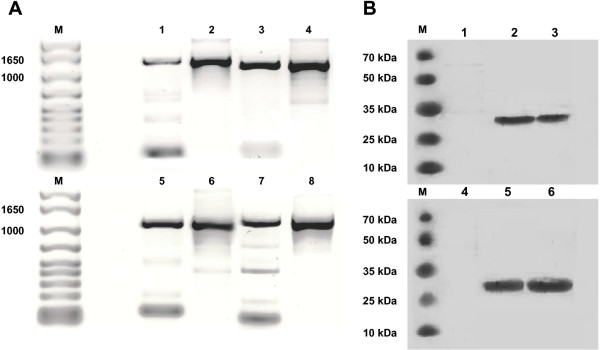
**Genetic stability of the viral constructs after five passages in chicken embryos (CE), as determined by RT-PCR (A) and confirmation of expression of the *Brucella *proteins L7/L12 or Omp16 by the viral constructs using Western blotting (B).** Figure 1A: 1) Flu-NS1-124-Omp16-H5N1; 2) pHW plasmid encoding the NS1-124-Omp16 genes; 3) Flu-NS1-124-Omp16-H1N1; 4) Plasmid NS1-124-Omp16; 5) Flu-NS1-124-L7/L12-H5N1; 6) Plasmid NS1-124- L7/L12; 7) Flu-NS1-124-L7/L12-H1N1; 8) Plasmid NS1-124- L7/L12. Figure 1В: 1) lysed allantoic fluid (AF) of uninfected CE; 2) lysed AF of CE infected with Flu-NS1-124-Omp16-H5N1; 3) lysed AF of CE infected with Flu-NS1-124-Omp16-H1N1; 4) lysed AF of uninfected CE; 5) lysed AF of CE infected with Flu-NS1-124- L7/L12-H5N1; 6) lysed AF of CE infected with Flu-NS1-124- L7/L12-H5N1.

Four recombinant influenza A viral constructs of the subtypes Н5N1 or H1N1 expressing the *Brucella* proteins L7/L12 or Omp16 were obtained by a reverse genetics method: Flu-NS1-124-L7/L12-H5N1, Flu-NS1-124-Omp16-H5N1, Flu-NS1-124-L7/L12-H1N1 and Flu-NS1-124-Omp16-H1N1. All of the viral constructs replicated well in embryonated chicken eggs (CE). It should be noted that during the initial passages in CE, the viral constructs had low infection and hemagglutination titers; however, as the number of passages increased, the titers also increased (Table 1). By the fifth passage, the infectious titers of the viral constructs ranged from 7.95 ± 0.22 to 9.2 ± 0.14 Log_10_ EID_50_/ml. Examination of the NS1 gene by RT-PCR confirmed that all of the viral constructs retained their *Brucella* inserts in CE over 5 passages (Figure 1A). The sizes of the NS1 genes of the viral constructs containing *Brucella* proteins L7/L12 or Omp16 corresponded to the size of that amplified from the control pHW plasmids (1110 and 1242 bp, respectively). These results were confirmed by the sequencing data, which showed that the nucleotide sequences of the NS1 gene of all of the viral constructs corresponded to the *Brucella* proteins L7/L12 or Omp16 (data not shown). Western blotting (Figure 1B) demonstrated that the fusion proteins NS1-L7/L12 and NS1-Omp16, with molecular masses of approximately 27 kDa and 33 kDa, respectively, were correctly expressed in CE infected with the viral constructs.

**Table 1:** Infection and hemagglutination titers for the viral constructs during passage in chicken embryos (CE)

**Passage level/biological system**	**Infection (Log**_**10 **_**EID**_**50**_**/ml) /hemagglutination titer**			
	**Flu-NS1-124-L7/L12-H5N1**	**Flu-NS1-124-Omp16-H5N1**	**Flu-NS1-124-L7/L12-H1N1**	**Flu-NS1-124-Omp16-H1N1**
1/CE	7.28 ± 0.3/1:128	6.82 ± 0.14/1:16	4.45 ± 0.14/1:128	4.2 ± 0.08/1:4
3/CE	8.78 ± 0.14/1:256	7.95 ± 0.14/1:256	7.95 ± 0.22/1:128	6.95 ± 0.14/1:256
5/CE	8.95 ± 0.22/1:512	8.45 ± 0.08/1:512	9.2 ± 0.14/1:512	7.95 ± 0.22/1:256

### Safety in mice

The safety or degree of attenuation of the viral constructs was determined in mice using three routes of administration, according to the methodology described in the Methods section. No animals in any group died during the entire observation period. The overall condition of the animals in the experimental and control groups was identical, in terms of physical activity, appetite and outward appearance.

Analysis of weight changes in the mice over 28 days after the prime or boost vaccinations showed an increase in the animals’ body weight, regardless of the type of viral construct or route of administration (data not shown). Mice in the experimental groups showed a similar increase in body weight (about 3.9-4.8 g or 23.6-29.0%) to the control group (4.7 g or 29.1%) by the end of the observation period.

Morphological and histological examinations of mouse organs (heart, lungs, liver, kidneys and spleen) 5 days after the prime or boost vaccinations revealed an absence of macro- and microscopic pathological changes (data not shown).

### Evaluation of virus shedding from upper-respiratory tract of the vaccinated guinea pigs

Titration results of nasal fluids in CE revealed that all guinea pigs vaccinated with mono and bivalent vaccine formulation by intranasal (*i.n.*) method did not shed the vaccine viruses through the upper respiratory tract in an observation period of 7 days after the prime and booster vaccination (data not shown).

### Antibody response against the Brucella L7/L12 and Omp16 proteins in immunized mice

**Figure 2:**
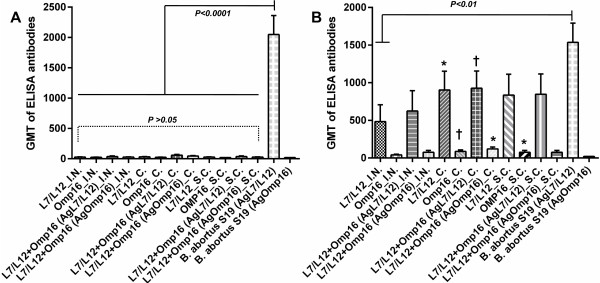
**Titers of antibodies against the L7/L12 and Omp16 proteins in mice from the experimental and control (on 28 [A] and 56 [B] days after a single vaccination with *B. abortus 19 *or administration of PBS) groups 28 days after prime (A) and boost (B) immunization by different routes of administration with mono- or bivalent vaccine formulations of the subtypes Н5N1 and Н1N1, as determined by ELISA.** Data for the control group is not shown; *i.n*. – intranasally, *c.* – conjunctivally*, s.c.* – subcutaneously; Ag – antigen. The data are presented as geometric mean titer (GMT) ± standard error (SE); * *P* < 0.05, † *P* < 0.01 compared to single vaccination (*n* = 5 mice per group). Statistical analysis was performed using a one way ANOVA followed by Tukey’s multiple comparisons test.

Blood samples were collected from mice of the experimental and control groups 28 days after the prime (D 28) and boost (D 56) immunizations to detect antibodies against L7/L12 and Omp16 using an enzyme-linked immunosorbent assay (ELISA). Single immunization with the mono- and bivalent vaccine formulations Flu-NS1-124-L7/L12-H5N1 and Flu-NS1-124-Omp16-H5N1 did not elicit significant (*P > 0.05*) *Brucella* antibody titers (Figure 2). L7/L12 and Omp16-specific IgG, of 52.7 ± 10.7 and 40.0 ± 6.4, respectively were observed only in one group of animals immunized by conjunctival (*c*.) method with the bivalent vaccine formulation. In contrast to experimental mice, mice of the positive control group vaccinated once with *B. abortus 19* developed at study day 28 a significant (*P <0.0001*) L7/L12-specific IgG response (2048 ± 313), but no Omp16-specific IgG.

An increase in the geometric mean titer (GMT) of antibodies against L7/L12 (9.3 –18.5-fold) and Omp16 (1.5-3.6-fold) for all tested routes of administration was observed after the booster vaccination in the experimental groups of mice immunized with the mono- and bivalent vaccine formulations. However, the most significant (*Р < 0.05-0.01*) increase in the GMT of *Brucella* antibodies after the booster vaccination was primarily observed in the groups of *c.* immunized mice. A slight decrease (*P > 0.24*) of L7/L12-specifc IgG (1536 ± 256) was determined on day 56 in mice vaccinated once with *B. abortus 19* when compared with the data at day 28 after immunization. It should be noted that antibody titers against L7/L12 in animals from experimental and positive control groups within the specified period of vaccination had no significant (*P > 0.05*) difference (except for the group of mice vaccinated *i.n.* with monovalent viral construct Flu-NS1-124-L7/L12, P <0.01).

### Evaluation of the T-cell immune response elicited by the viral constructs

**Figure 3:**
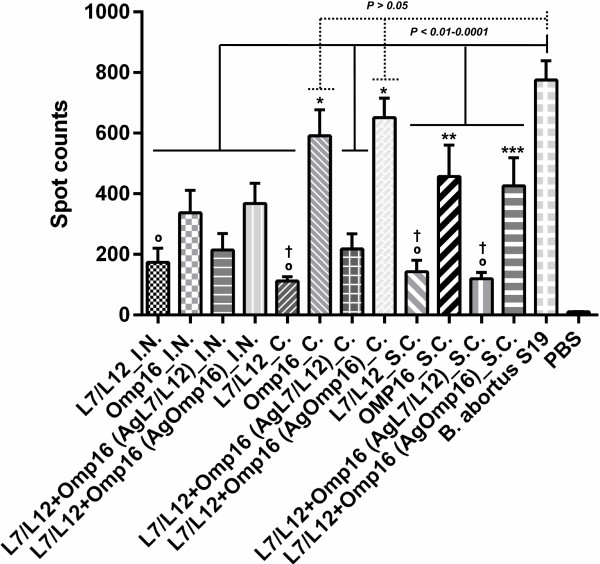
**Quantitative ELISPOT analysis of IFN-γ-producing lymphocytes in immunized mice after *in vitro *stimulation of isolated splenocytes (4 x 10**^**5**^**cells) with the *Brucella *proteins L7/L12 or Omp16 or heat-inactivated *B. abortus 19 *(10**^**6**^ **CFU).** The mice were immunized with mono- and bivalent vaccine formulations of the subtypes Н5N1 or H1N1 by prime-boost intranasal (*i.n*.), conjunctival (*c.*) *or* subcutaneous (*s.c.*) vaccination 28 days apart, and with *B. abortus 19* by single *s.c.* vaccination. Mice from the negative control groups were *s.c.* administered with PBS. Quantitative spot ± SE values (3 replicates per mouse) are presented for each group of 5 mice. *Р < 0.005 or *Р* < 0.0001 compared to the all group of animals vaccinated with the Flu-NS1-124-L7/L12 (Н5N1 + H1N1) viral constructs; **Р < 0.05 or *Р* < 0.025 compared with º; ***Р < 0.05 or *Р* < 0.025 compared with †. Statistical analysis was performed using a one way ANOVA followed by Tukey’s multiple comparisons test.

The degree of *Brucella*-specific cellular immunity induced in mice by the mono- and bivalent vaccine formulations expressing the L7/L12 or Omp16 proteins via different routes of administration or by *B. abortus 19* (D 56) was determined using an enzyme-linked immunospot assay (ELISPOT) assay 28 days after the boost vaccination. Compared to the control group (PBS), the mono- and bivalent vaccine formulations administered by prime-boost vaccination elicited a strong T-cell immune response (*Р* < 0.005 or *Р* < 0.001), regardless of the route of administration, as indicated by the presence of a large number of IFN-γ-producing lymphocytes (ranging from 112 ± 9 to 651 ± 45 spots/4 × 10^5^ cells; Figure 3). Compared to the group of animals vaccinated with the Flu-NS1-124-L7/L12 (Н5N1 + H1N1) viral constructs, the number of spots were significantly higher for the groups of mice vaccinated with the viral vectors encoding the *Brucella* Omp16 protein, especially when administered by the *c.* (*Р* < 0.005 or *Р* < 0.0001) and subcutaneous (*s.c.*; *Р* < 0.05 or *Р* < 0.025) routes. There was no significant difference (*Р* > 0.05) in the number of spots for the individual viral constructs expressing the L7/L12 protein or Omp16 protein for the different routes of administration. Among all of the experimental groups of mice only two groups which were immunized by *c.* route with viral vectors encoding the *Brucella* Omp16 protein induced comparable number of spots (P > 0.05) than did mice of the positive control group vaccinated with *B. abortus 19*. Spot numbers in remaining experimental groups were significantly lower (*Р* < 0.01 - *Р* < 0.0001).

### Study of protective efficacy

The protective efficacy of the mono- and bivalent vaccine formulations was investigated in a guinea pig model and compared to a commercial live vaccine produced from strain *B. abortus* 19, according to the methodology described in the Methods section. The protective efficacy of the vaccines was evaluated using parameters such as the effectiveness of vaccination, index of infection (I.I. is the number of organs and lymph nodes from which *Brucella* bacteria were isolated), and bacterial load of the virulent *B. abortus* challenge strain 544 in spleens of vaccinated and unvaccinated (PBS) animals.

**Figure 4:**
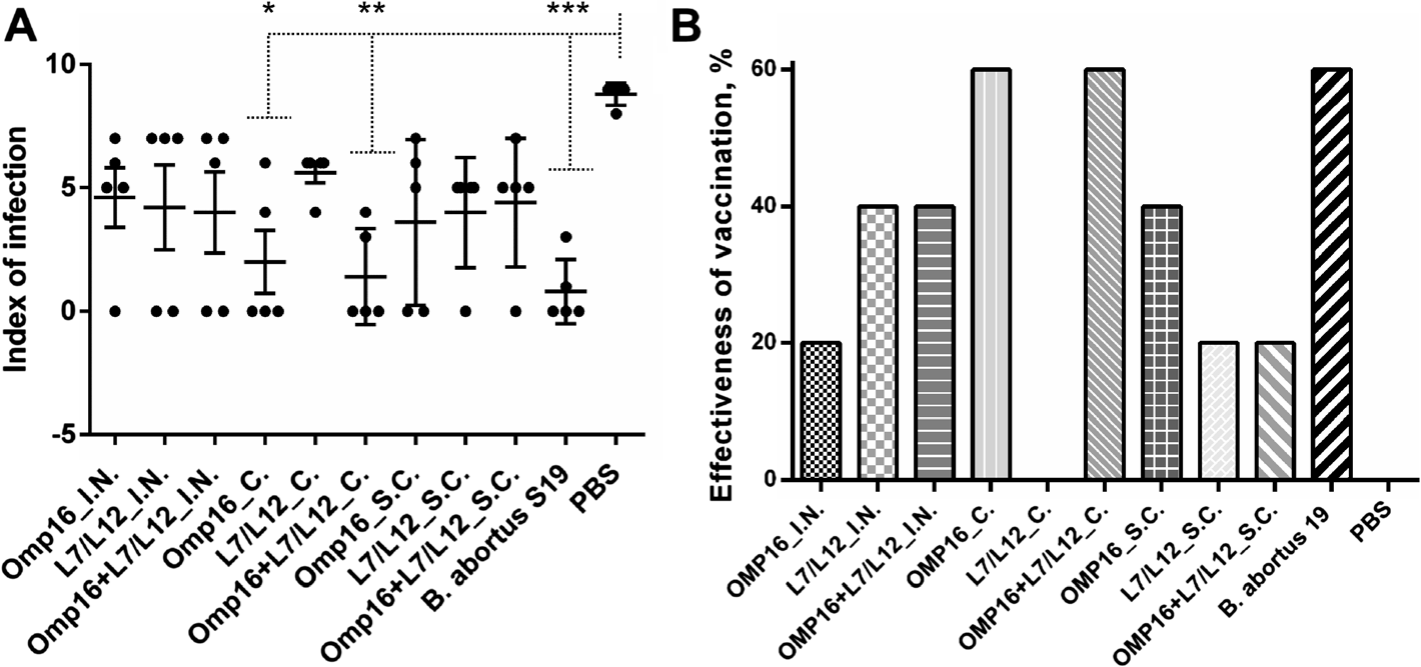
**Protective efficacy of the vaccines in a guinea pig model when administered by different routes, as evaluated by index of infection (A; the number of organs and lymph nodes from which *Brucella *was isolated) and effectiveness of vaccination (B).** The guinea pig were immunized with mono- and bivalent vaccine formulations of the subtypes Н5N1 or H1N1 by prime-boost intranasal (*i.n*.), conjunctival (*c.*) *or* subcutaneous (*s.c.*) vaccination 28 days apart, and with *B. abortus 19* by single *s.c.* vaccination. Guinea pigs of the negative control groups were obtained *s.c.* of PBS. * *P < 0.005*, ** *P < 0.001*, *** *P < 0.0006* compared to negative control groups. Statistical analysis was performed using a one way ANOVA followed by Tukey’s multiple comparisons test.

Compared to the control (PBS) group, all of the vaccines, regardless of the route of administration, provided significant protection (*Р* < 0.01 - *Р* < 0.001) of the guinea pigs against infection by the strain *B. abortus* 544 to some extent, in terms of bacterial load of the virulent strain in spleens of animals after challenge (Table 2). According to the I.I. (Figure 4) significant protection compared to the control (PBS) group was achieved only in the animals that were *c.* vaccinated with monovalent viral constructs expressing the Omp16 protein (*P < 0.005*; effectiveness of vaccination 60%) and bivalent vaccine formulation expressing the L7/L12 and Omp16 proteins (*P < 0.001*; effectiveness of vaccination 60%), and also in the animals vaccinated with *B. abortus* 19 (*P < 0.0006*; effectiveness of vaccination 60%). It should be emphasized that the I.I. in animal groups vaccinated with the above-mentioned viral constructs were similar (*Р* > 0.99) to those vaccinated with the commercial *B. abortus* 19 vaccine.

**Table 2:** **Degree of protective efficacy of the vaccines as evaluated by the isolation rate of *Brucella *** **from the spleens of guinea pigs challenged with the virulent strain *B. abortus *** **544**

**Vaccine**	**Route of administration**	**Log**_**10 **_**CFU/spleen (mean ± SE)**	**Log**_**10 **_**protection**	*Value (*P*)
Monovalent Flu-NS1-124-Omp 16 (H5 + H1)	*i.n.*	2.0 ± 0.52	2.54	<0.01
	*c.*	0.76 ± 0.44	3.78	<0.001
	*s.c.*	1.26 ± 0.52	3.28	<0.005
Monovalent Flu-NS1-124-L7L12 (H5 + H1)	*i.n.*	1.22 ± 0.50	3.32	<0.001
	*c.*	2.4 ± 0.25	2.14	<0.005
	*s.c.*	1.46 ± 0.39	3.08	<0.001
Bivalent vaccine formulation Flu-NS1-124-Omp 16 + Flu-NS1-124-L7L12 (H5 + H1)	*i.n.*	1.28 ± 0.52	3.26	<0.005
	*c.*	0.64 ± 0.40	3.90	<0.001
	*s.c.*	1.68 ± 0.51	2.86	<0.005
*B. abortus* 19	*s.c.*	0.42 ± 0.26	4.12	<0.001
Control (PBS)	*s.c.*	4.54 ± 0.43	0.00	

*i.n*. – intranasally, *c.* – conjunctivally*, s.c.* – subcutaneously; *compared with control group. Statistical analysis was performed using a one way ANOVA followed by Tukey’s multiple comparisons test.

## Discussion

The currently available commercial live *Brucella* vaccines have serious intrinsic drawbacks, which is why vaccination is restricted in many countries prone to brucellosis. This situation has motivated many researchers to develop new generations of vaccines which do not possess such drawbacks, but which can induce the same protection efficacy as commercial vaccines.

In our opinion, of the strategies currently being proposed, live vector vaccines are the most likely replacement for existing commercial vaccines against bovine brucellosis, given the long-term experience of their successful use in veterinary practice [30].

In this study, we first developed recombinant influenza A viruses of the subtypes Н5N1 and H1N1 expressing *Brucella* ribosomal protein L7/L12 or Omp16, and then demonstrated that these vaccines could be used as new candidates for a brucellosis vaccine. The choice of the *Brucella* proteins L7/L12 and Omp16 as antigenic determinants was based on their immunodominance in eliciting Th1 CD4^+^ and CD8^+^ T-cell immune responses [8-10,13,14,16,20,22]. In addition, it was previously shown that these recombinant proteins, both when pure, combined with adjuvants, or expressed by DNA vaccines, provided mice with good protection when challenged with virulent *B. abortus* 544 [8,10,13,14,20]*.*

A large body of data [31-34] has confirmed the ability of influenza viruses to infect cattle and elicit a serological reaction and, in some cases clinical disease, which provided the basis for choosing influenza A viruses as the vaccine vector in this study. Thus, the attenuated influenza A viruses selected as the vector should be able to infect cattle and express the recombinant *Brucella* proteins. The potential of the influenza A NS vector was confirmed in our previous studies of the development of a tuberculosis vaccine [35].

On the basis of previous study [36], and in order to achieve maximum expression of the *Brucella* proteins *in vivo* and elicit an increased T-cell immune response, the laboratory animals were immunized using a double vaccination schedule with viral constructs of the Н5N1 subtype (prime vaccination) and H1N1 subtype (booster vaccination). This immunization strategy effectively overcomes the immune background elicited against the viral vector during primary vaccination.

Since influenza viruses are primarily transmitted within droplets, and reproduce in the mucous membranes of the respiratory tract, the *i.n*. route was chosen for immunizing the animals, and the *c.* and *s.c*. routes were used for comparison. The mucosal routes of administration (*i.n.* and *c.*) were chosen as the mucous membranes are the main gateway for brucellosis infection, and these routes have been shown to provide immunized animals with a high level of protection against the virulent *B. abortus* strain [13,37].

The first series of experiments established that despite the truncated nature of the NS1 gene, the viral constructs Flu-NS1-124-L7/L12-H5N1, Flu-NS1-124-Omp16-H5N1, Flu-NS1-124-L7/L12-H1N1, and Flu-NS1-124-Omp16-H1N1 had good reproductive properties in CE over five consecutive passages and retained their *Brucella* inserts.

In our previous studies, it was shown that as the size of the NS1 gene decreased in viral vectors, the degree of attenuation of the influenza A viruses increased [38]; however, it is well known that attenuation of influenza viruses may be dependent on the properties of the foreign insert in the C-terminal part of the truncated NS1 protein [39]. Therefore, we considered it necessary to study the safety or degree of attenuation of the constructed recombinant influenza A viruses in mice, which provide the most sensitive model for testing the safety of influenza vectors. Both the mono- and bivalent vaccine formulations containing *Brucella* inserts (L7/L12 or Omp16) in the *NS1* gene were safe in mice when administered by the *i.n., c.* or *s.c.* routes. No deaths, loss of body weight or pathomorphological changes occurred in the mice over the entire observation period, which provided evidence for the attenuation of the influenza A viruses.

Moreover it has to be noted that all guinea pigs vaccinated with mono and bivalent vaccine formulation by *i.n.* method did not shed the vaccine viruses through their upper respiratory tract the entire week after the prime and booster vaccination. These results proved the replication-deficient properties of the virus vectors and confirm no danger of viral transmission from vaccinated to non-vaccinated animals or people. Interestingly, one vaccine vector is based on the prepandemic flu A/H5N1 delNS1 vaccine. It was shown previously that this vaccine is completely safe and immunogenic when tested in a variety of laboratory models (chickens, ferrets and rhesus macaques) [40] and humans [41]. We can assume that this vaccine in the future can be used not only for cattle but also for humans.

Furthermore, the mono- and bivalent vaccine formulations of the subtypes Н5N1 and H1N1 elicited antigen-specific humoral and T-cell immune responses after prime-boost immunization via all of the tested routes of administration. The greatest antibody response (by ELISA) was obtained with the viral constructs expressing the *Brucella* protein L7/L12; and the greatest T-cell immune response by ELISPOT assay was obtained with viral constructs expressing the Omp16 protein (*c.* immunization), not inferior (*P > 0.05*) to commercial vaccine *B. abortus 19*. It should be noted that, as the humoral and cellular immune responses indicate, the bivalent vaccine formulation were in no way inferior (*P > 0.05*) to their monovalent variants, despite the lower (by half) dose injected; the bivalent vaccine formulation even somewhat surpassed their monovalent variants. We also demonstrated that single or combined injection of mice with viral constructs expressing two different *Brucella* proteins did not lead to interference between the constructs, as the mice immunized with bivalent vaccine formulation formed a humoral and cellular immune responses to both (L7/L12 or Omp16) protein of *B. abortus*. The optimal T-cell immune responses were achieved by the *c.* immunization route, despite the fact that influenza viruses primarily reproduce in respiratory tract organs. In our opinion, this is mainly due to the ability of influenza viruses to reproduce not only in the conjunctival mucous membrane, but also in the cells of the cornea thus priming the conjunctiva-associated lymphoid tissue (CALT) and eye-associated lymphoid tissue (EALT). CALT can detect antigens from the ocular surface, present the antigens and generate protective effector cells; together, these properties signify the presence of a mucosal immune system at the conjunctiva [42-44]. Theoretically, the administration of antigens into the conjunctival sac would additionally drain to the nasal-associated lymphoid tissue (NALT). It was shown previously that conjunctival or intraocular infection with influenza viruses stimulates the local as well as systemic immune response [45]. Another explanation for the higher cellular immunity obtained using viral constructs expressing Omp16 is that it Omp16 is able to act as an adjuvant and activate dendritic cells and macrophages *in vitro*, according to Pasquevich et al. [46].

Data in mice have found a logical reflection in experiments with guinea pigs. Guinea pigs were chosen as model animals for studying the protective efficacy of the viral constructs in this study due to their higher resistance to influenza infection than mice [35]. In this case, the use of a more resistant animal model was a key condition for studying protective efficacy, since the vaccine is ultimately intended for cattle. Furthermore, according to Silva et al., guinea pigs are the most susceptible model for evaluating protective efficacy [28] for the commercial live brucellosis vaccine produced from strain *B. abortus* 19, This strain was used as a reference in the present study. The protective efficacy of the vaccines was evaluated by assessing both the bacterial load of the virulent strain *B. abortus* 544 in spleens of vaccinated and unvaccinated animals, and by other parameters such as the effectiveness of vaccination and I.I. In our opinion, when taken together, these parameters provide a more complete and objective characterization of the protective efficacy of vaccines.

In this study, guinea pigs were challenged with *B. abortus* 544 at a dose of 5 × 10 CFU/animal, while in other similar studies, mice were used as the model animals and higher doses of 4 × 10^4^ - 5 × 10^5^ CFU were administered [6,13,20]. Our previous studies (unpublished data) showed that *s.c.* administration of five *B. abortus* 544 microbial cells caused generalized infection (minimum infective dose) in guinea pigs. On this basis, a dose of ten times the minimum infective dose, i.e., 5 × 10 CFU/animal, was used to evaluate the protective efficacy of the vaccines. This dose enabled a more objective comparative evaluation of the protective efficacy of the vaccines.

In terms of the effectiveness of vaccination, I.I., and isolation rate, the highest levels of protection were achieved in the guinea pigs which were *c.* vaccinated with monovalent viral constructs expressing the Omp16 protein and bivalent vaccine formulation expressing the L7/L12 and Omp16 proteins. It is noteworthy that the groups revealing the highest protective efficacy also induced also the best cell-mediated immunity. These experiments demonstrated that the monovalent viral constructs expressing the Omp16 protein and bivalent vaccine formulation expressing the L7/L12 and Omp16 proteins, when administered in prime-boost *c.* immunization mode, were comparable in terms vaccine effectiveness and protective efficacy to the commercial live vaccine produced from strain *B. abortus* 19 in guinea pigs. It should be noted that the results obtained in the present study was successfully used in evaluating the immunogenicity and efficacy of our vector vaccine in cattle. It has been established that the administration of the vector vaccine via the *c.* method of vaccination promoted formation of IgG antibodies (with a predominance of antibodies of isotype IgG2a) in cattle against *Brucella* L7/L12 and Omp16 proteins in ELISA. Moreover, these vaccines in cattle induced a strong antigen-specific T-cell immune response and provided a high level of protection efficacy comparable to those of the commercial *B. abortus S19* vaccine [47].

## Conclusions

Thus, we conclude that administration of recombinant influenza A viruses of the Н5N1 and H1N1 subtypes expressing L7/L12 or Omp16 in prime-boost *i.n., c.* or *s.c.* immunization modes is safe; induce antigen-specific humoral and T-cell immune responses in laboratory animals, and, most importantly, provide a high degree of protective efficacy which is comparable to that offered by a commercial live vaccine produced from strain *B. abortus* 19. On this basis, we propose that this recombinant influenza A viruses represent a novel, effective candidate vaccine against *B. abortus*.

## Methods

### Cells

The Vero (kidney epithelial cells isolated from an African green monkey) and MDCK (Madin-Darby Canine Kidney) cell lines originated from the American Type Culture Collection (Manassas, VA, USA). Vero adapted to grow in serum-free medium were maintained in a serum-free OPTIPRO medium (GIBCO). MDCK cells were cultivated in a 1:1 mixture of DMEM and Ham’s F12 medium containing 2% heat inactivated fetal bovine serum (FBS; SH30071; HyClone, Logan, UT, USA) and 4 mM *L-*glutamine.

### Bacterial strains

The vaccine strain *B. abortus* 19 (Shchelkovsky Biokombinat, Moscow oblast, Russia) and the virulent strain *B. abortus* 544 (obtained from our institute’s collection of microorganisms) were used in this study. The bacterial cells were cultured under aerobic conditions in tryptone soy agar (TSA; Sigma, St. Louis, MO, USA) at 37°C. All experiments with live *Brucella* were performed in biosafety level 3 facilities. *Escherichia coli* strain T7 (New England Biolabs, Frankfurt am Main, Deutschland, UK) was used to prepare the recombinant proteins. The *E. coli* cultures were routinely grown at 37°C in Luria-Bertani (LB) broth or agar which was supplemented, when required, with 100 μg/ml ampicillin.

### Generation of viruses

All viruses were generated by a standard reverse genetics method using 8 bidirectional plasmids pHW2000 [48]. Briefly, Vero cells were co-transfected by LonzaNucleofector™ (Cologne, Germany) technique with 0.5 μg/μl of plasmids encoding the PB1, PB2, PA, NP, M gens and NS (chimeric) gene of А/Puerto Rico/8/34 (H1N1) virus; and the HA and NA genes of A/chicken/Astana/6/05 (H5N1) or А/New Caledonia/20/99 (H1N1) strains. The HA protein sequence of the H5 virus was attenuated by means of exchanging its polybasic cleavage site to one containing a trypsin-dependent sequence. The NS genes were modified to express NS1 fusion proteins containing a sequence of 124 N-terminal amino acids from the NS1 protein coupled with a sequences of *B. abortus* derived proteins: L7/L12 (GenBank: AAA19863.1) or Omp16 (GenBank: AAA59360.1), ended with double stop codon. *Brucella* sequences were obtained synthetically. The supernatants of transfected cells were used for inoculation into 10-day-old CE (Lohmann Tierzucht GmbH, Cuxhaven, Germany]) which was incubated at 34°С for 48 h. Vaccine batches were produced in CE after three egg passages of viral constructs.

### Genetic stability of the viral constructs

Five consecutive passages of four of the viral constructs were accumulated in 10-day-old CE for genetic stability testing. The allantoic cavity of the CE were infected using 10^−4^ dilutions of inoculate. The genetic stability of the viral constructs was confirmed by reverse transcription-polymerase chain reaction (RT-PCR [49]) using NS segment specific primers (sense 5′-ACTACTTCTAGAGAAGACAAAGCAAAAGCAGGGTGACA-3′ and antisense 5′-ACTACTCTGCAGATTAACCC TCACTAAAAGTAGAAACAAG-3′). At passages 1, 3 and 5; the size of the NS amplicons was compared to those of pHW plasmids encoding the corresponding genes. The NS1*-*fusion protein encoding genes of the viral constructs were sequenced at passages 1, 3 and 5 using the Sanger method with the commercial kit Prism BigDye™ Terminator v3.1 (ABI, Foster City, CA, USA) on a automatic 16-capillary sequencer Genetic Analyser 3130 ×l (ABI).

### Determination of the infectious titers of the viruses

The infectious titers of the viruses were determined using 10-day old specific pathogen free (SPF) embryonated chicken eggs by standard methods. Viral suspensions (10^−1^ – 10^−9^ dilutions) were prepared in PBS (pH 7.2) and the allantoic cavities of four CE were infected with 0.2 ml of each dilution. The CE were incubated at 34 ± 0.5°C at a relative humidity of 60 ± 5% for 48 h. After cooling by the drop method, the viral titers were determined by the hemagglutination assay [50]. Viral titers were calculated using the method of Reed and Muench [51], and expressed as Log_10_ EID_50_/ml.

### Production of recombinant Brucella proteins

In order to obtain the recombinant L7/L12 and Omp16 proteins, the nucleotide sequences of the genes were amplified from the genomic DNA of *B. abortus* 19 and cloned into рЕТ (Novagen, Darmstadt, Germany) plasmid vectors, bacterial DNA was isolated using PrepManUltra kits (ABI). The primers used to amplify the genes are listed in Table 3. Amplification was performed in 50 μl reactions containing 5 μl of 10× PCR buffer (Qiagen, Hilden, Germany), 1 μl of 10 mM dNTPs (NEB), 0.1 μl DNA, 1 μl of each primer (20 pmol/μl), and 0.25 μl *Taq* DNA polymerase (2.5 U; Qiagen). Amplification conditions were as follows: 94°C for 5 min; 30 cycles of 94°C for 1 min, 50°C for 1 min and 72°C for 2 min; and a final extension at 72°C for 7 min. The amplified PCR products of the L7/L12 and Omp16 genes were cloned into the рЕТ26 expression vector using the *Xho*I and *Nde*I sites; the recombinant L7/L12 protein contained a 6-HIS tag at the N- and C-termini, and the recombinant Omp16 protein contained a 6-HIS tag at the C-terminus. Next, *E. coli* strain T7 cells were transformed with the recombinant expression plasmids, and the recombinant proteins were purified using Ni-NTA Agarose (MCLAB, South San Francisco, CA, USA) according to the manufacturer’s instructions. The purified proteins were identified by sodium dodecyl sulfate-polyacrylamide gel electrophoresis (SDS-PAGE) and Western blot assays. The purified protein was stored at – 70°C until use for ELISA or for in vitro stimulation of splenocytes.

**Table 3:** **Primers used for amplifying genes encoding *Brucella *** **proteins**

**Gene**	**Primer**	**Sequence**
*Omp16* (GenBank ID: AAA59360)	omp16-f	5′- CG**CAT** ***ATG*** CGCCGTATCCAGTCGATTGCA -3′
	omp16-r	5′- CG**CTCGAG**CCTTCCGGCCCCGTTGAGAA -3′
*L7/L12* (GenBank ID: AAA19863.1)	L7/L12-f	5′-CG**CAT** ***ATG*** GGAATTTCAAAAGCAAGTCT-3′
	L7/L12-r	5′-CG**CTCGAG**GCGCGACAGCGTCACGGCCT-3′

Restriction sites are bold-faced, and start codons are shown in italics.

### Western blot analysis of the expression of Brucella proteins

Samples of allantoic fluid from uninfected CE and CE infected with the viral constructs Flu-NS1-124-L7/L12-H5N1, Flu-NS1-124-Omp16-H5N1, Flu-NS1-124-L7/L12-H1N1, or Flu-NS1-124-Omp16-H1N1 were mixed with RIPA lysis buffer (Sigma) in a 1:1 ratio, heated at 100°С for 5 min and centrifuged for 5 min at 13,000 rpm. Electrophoretic separation of the proteins was performed using 12% SDS-PAGE gels, and Coomassie G-250 dye or Western blotting was used to visualize the proteins. For Western blotting, the proteins were transferred to nitrocellulose membranes. The membranes were blocked at room temperature for 1 h in blocking buffer (150 mМ NaCl, 20 mM Tris–HCl, pH 7.5, containing 5% skimmed milk powder). The blot was probed with rabbit anti L7/L12 or Omp16 serum (Eurogentec S.A., Belgium) at a dilution 1:2000 in blocking buffer containing 0.1% Tween-20 for 2 h at room temperature. Following 3 washes with TBST buffer (150 mM NaCl, 20 mM Tris–HCl, pH 7.5, 0.1% Tween-20), the blot was incubated for 1 – 2 h at room temperature in G-alkaline phosphatase-conjugated goat-anti-rabbit IgG antibody (Sigma) [8]. The membranes were washed three times for 10 min each with TBST buffer, and then the bands were visualized by the addition of BCIP/NBT substrate (Sigma).

### Laboratory animals and bioethics

This study used 150 female BALB/c mice aged 6-8-weeks-old weighing 15 to 18 g (Charles River Laboratories, Erkrath, Germany) and 55 female outbreed guinea pigs weighing 300–350 g (National Center for Expertise of Drugs, Medical Products and Equipment, Almaty, Kazakhstan). The laboratory mice were randomly divided into 11 groups: nine experimental prime-boost groups (*n* = 14 per group) immunized with the mono- or bivalent vaccine formulations either *i.n*. (*n* = 42), *c*. (*n* = 42) or *s.c*. (*n* = 42), one negative (PBS) control group (*s.c*., *n* = 14), and one positive (*B. abortus* 19) control group (*s.c*., *n* = 10). The guinea pigs were divided into 11 groups: nine experimental (*n* = 5 per group) prime-boost groups immunized with the mono- or bivalent vaccine formulations *i.n*. (*n* = 15), *c.* (*n* = 15) or *s.c*. (*n* =15), one negative (PBS) control group (*s.c*., *n* = 5), and one positive control group vaccinated with *B. abortus* 19 (*s.c*., *n* = 5). The absence of outward signs of disease and homogeneity of the groups by body weight (±20%) were used as eligibility criteria for randomization. Experimental and control groups of animals were kept in different rooms during the entire experiment and all animals had free access to water and standard rodent diet.

This study was carried out in compliance with national and international laws and guidelines on laboratory animal handling. The protocol was approved by the Committee on the Ethics of Animal Experiments of the Research Institute for Biological Safety Problems Science Committee of the Ministry of Education and Science of the Republic of Kazakhstan (Permit Number: 1012/405).

### Immunization

After light anesthesia with methoxyflurane (Abbott Laboratories, Abbot Park, Illinois, USA), the mice and guinea pigs were *i.n*., *c.* or *s.c*. immunized twice with recombinant influenza A viruses of the Н5N1 subtype (prime vaccination) and H1N1 subtype (booster vaccination) 28 days apart. A detailed immunization chart for the animals is shown in Table 4. Guinea pigs and mice from the positive control group were immunized once *s.c*. with the attenuated strain *B. abortus* 19 at a dose of 2 × 10^9^ CFU/animal and 10^5^ CFU/animal, respectively. Mice and guinea pigs of the negative control groups were obtained *s.c.* 200 μl of PBS.

**Table 4:** Scheme of immunization of animals with recombinant influenza A viruses of the subtypes Н5N1 and H1N1

**Species**	**Viral construct**	**Route of administration**	**Number of animals**	**Prime vaccination dose (Н5N1), Log**_**10 **_**EID**_**50**_**/animal**	**Boost vaccination dose (Н1N1), Log**_**10 **_**EID**_**50**_**/animal**
Mice	Monovalent Flu-NS1-124-L7/L12	*i.n.*	10	5.97	6.39
		*c.*	10	5.58	6.08
		*s.c.*	10	6.58	7.00
	Monovalent Flu-NS1-124-Omp16	*i.n.*	10	5.51	5.64
		*c.*	10	5.20	5.33
		*s.c.*	10	6.12	6.25
	Bivalent vaccine formulation Flu-NS1-124-L7/L12 + Flu-NS1-124-Omp16	*i.n.*	10	5.58 + 5.20	6.08 + 5.33
		*c.*	10	5.27 + 4.89	5.69 + 4.99
		*s.c.*	10	6.27 + 5.81	6.69 + 5.94
Guinea pigs	Monovalent Flu-NS1-124-L7/L12	*i.n.*	5	6.28	6.70
		*c.*	5	5.89	6.39
		*s.c.*	5	6.58	7.00
	Monovalent Flu-NS1-124-Omp16	*i.n.*	5	5.82	5.95
		*c.*	5	5.51	5.64
		*s.c.*	5	6.12	6.25
	Bivalent vaccine formulation Flu-NS1-124-L7/L12 + Flu-NS1-124-Omp16	*i.n.*	5	5.97 + 5.51	6.39 + 5.64
		*c.*	5	5.58 + 5.20	6.00 + 5.30
		*s.c.*	5	6.27 + 5.81	6.69 + 5.94

The amounts of inoculate for the intranasal (*i.n*.), conjunctival (*c.*) or subcutaneous (*s.c.*) immunization routes were 50, 25, and 200 μl for mice, and 100, 50 and 200 μl for guinea pigs, respectively.

### Evaluation of the safety of the viral constructs in mice

The safety of the viral constructs (or their degree of attenuation) was evaluated in mice, and compared to the negative (PBS) control group. For this purpose, the vaccinated mice were observed daily. Safety was evaluated by monitoring the animals’ survival rate, overall condition, behavior and weight changes. Following necropsy morphological and histological examinations were performed.

The animals’ survival rate, overall condition and behavior after immunization were evaluated over a 56-day clinical observation period. The animals were weighed 0 and every day during 1–28 days after each vaccination.

### Sampling

To evaluate the safety of the vaccine vectors, two animals from each experimental and negative control group were euthanized by cervical dislocation 5 days after each vaccination (*n* = 36). The heart, lungs, liver, kidneys and spleen were excised for histological examination from the euthanized mice after a post-mortem pathological-anatomical examination. Twenty-eight days after the prime (D 28) and boost (D 56) vaccinations, five mice from each experimental, negative and positive control group were euthanized and the spleens were collected aseptically to determine the cellular immunity level using an ELISPOT. Blood samples were collected to identify antibodies against the *Brucella* proteins L7/L12 and Omp16 using ELISA. Nasal swabs were collected everyday during one week after each *i.n.* vaccination with mono – and bivalent vaccine formulations and eluted in tubes containing 1 ml of virus transport media (sterile solution of calf infusion broth, fraction V of bovine albumin, gentamicin sulphate, and Fungizone®) to evaluate the virus shedding. After brief centrifugation the nasal fluids were kept at - 70°C until titration in 10-day-old CE.

### Histological examination

The heart, lungs, liver, kidneys and spleen were collected from the immunized mice for histological examinations. Histological analysis was performed according to standard procedures using hematoxylin-eosin (Sintakon, St. Petersburg, Russia) stained sections.

### Isolation of lymphocyte cell populations and ELISPOT assay

Spleens were mechanically dissociated into single cell suspensions and filtered through cell strainers (BD Falcon, BD Biosciences, San Jose, CA, USA). Red blood cells were lysed using ACK solution (150 mM NH_4_Cl, 1 mM KHCO_3_, 0.1 mM Na_2_-EDTA, pH 7.3). Splenocytes were cultured at 37°C in 5% CO_2_ in 96-well flat-bottomed plates at an initial density of 4 × 10^5^ cells/well in RPMI-1640 medium supplemented with 2 mM *L-*glutamine and 10% heat-inactivated FCS (Sigma), in the presence of 0.8 μg of purified L7/L12 protein or Omp16 protein or heat-inactivated *B. abortus 19* (10^6^ CFU/well), or no additives (unstimulated control). To determine the numbers of cytokine-producing T cells, spleen cells were stimulated *in vitro* for 48 h with L7/L12 protein or Omp16 protein or heat-inactivated *B. abortus 19*, collected, and IFN-γ was detected by an ELISPOT assay utilizing Multiscreen-IP Millipore plates (Millipore, Bedford, MA). All assays were performed in triplicate. The spot number corresponding to the IFN-γ-producing cells was calculated using an ELISPOT spot counter (Biorader 4000 PRO-X; BIOSYS GmbH, Germany). Cells incubated in the absence of a stimulating antigen developed <15 spots/4 × 10^5^ cells.

### ELISA

Ninety-six well microtiter plates (Nunc, Roskilde, Denmark) were coated overnight with 2 μg/ml L7/L12 protein or Omp16 protein in PBS, blocked for 1 h using PBS-1% ovalbumin (PBS-OVA; 200 μl/well), and washed with PBS containing 0.05% Tween-20 (PBS/Tw). Serial two-fold dilutions of the serum samples from immunized mice (100 μl/well) diluted in PBS/OVA were added to the plates and incubated for 1 h at room temperature. After washing (4×), anti-rabbit IgG-HRP (100 μl of 1:2000/well, Dako, Cambridge, UK) was added and the plates were incubated for 1 h at room temperature. Following a washing step (4×), the plates were incubated with the substrate O-phenylenediamine (OPD; 100 μl/well; Norgen Biotek Corp., Ontario, Canada). The colorimetric reaction was stopped with 2.5 M H_2_SO_4_ (100 μl/well) and the optical density values were measured at 492 nm (reference wavelength, 620 nm). The cut-off value for titer determination was calculated based on the mean OD values of wells containing only buffer (blank) + three standard deviations. Endpoint serum ELISA titers are presented as GMT ± standard error (SE).

### Evaluation of protective efficacy

Twenty-eight days after the boost vaccination, guinea pigs from the experimental (*n* = 45) and negative (PBS) control groups were challenged *s.c.* with the virulent strain *B. abortus* 544 at a dose of 5 × 10^2^ CFU/animal. Guinea pigs from the positive control group (*n* = 5) were challenged in a similar manner 56 days after a single immunization with the vaccine strain *B. abortus* 19. Thirty days post-challenge, all of the guinea pigs were euthanized by CO_2_ asphyxiation and aseptically autopsied to remove the following organs: retropharyngeal, lower cervical, right and left inguinal and paraaortic lymph nodes, and liver, kidney, spleen, and bone marrow. The organs were plated onto TSA plates and the plates were incubated at 37°С for 4 weeks. During this time the growth of bacterial colonies was periodically counted. An animal was considered to be infected if a *Brucella* colony was detected from one or more organs. The results of the bacteriological examination were evaluated as the number of animals from which no colonies were isolated (effectiveness of vaccination) and by the index of infection (the number of organs and lymph nodes from which were isolated *Brucella*).

Determination of virulent *Brucella* from the spleens of challenged animals was used as an additional indicator of evaluation of protective efficacy. For this purpose, the collected spleens were homogenized in 2 ml of 0.1% Triton-PBS, and 100 μl aliquots of 10-fold serial dilutions were plated in triplicate onto TSA plates, incubated for 14 days at 37°C, and the number of CFU were counted. Log_10_ units of protection were calculated as the mean log_10_ numbers of CFU of the negative control group (PBS) minus the mean log_10_ number of CFU of the experimental group.

### Statistical analysis

The difference in antibody titers to Brucella L7/L12 and Omp16 proteins in ELISA and IFN-ץ producing cell in ELISPOT assay between the groups of mice depending on the vaccine formulation and methods of administration was performed using one way ANOVA followed by Tukey’s multiple comparisons test.

When defining a protective of mono-or bivalent vaccine formulations the difference between the experimental, positive and the negative control groups (PBS) in the number (log10 CFU) allocated *B. abortus* 544 from the spleen of guinea pigs and I.I. after challenge was determined using a one way ANOVA followed by Tukey’s multiple comparisons test.

*P* values < 0.05 were considered significant.

## Competing interests

KT, AS, ZhK, KS, BY, ShR, NZ, NA, NS, BKh are employees of the republican government enterprise on the basis of economic control rights «Research Institute for Biological Safety Problems» (RIBSP) of Science Committee of Ministry of Education and Science of the Republic of Kazakhstan. Other co-authors like IK, BF and AE are employees of a private company HSC Development GmbH, Tulln, Austria. The new recombinant influenza A H5N1 and H1N1 viruses expressing the *Brucella* ribosomal protein L7/L12 or outer membrane protein (Omp) - 16 is the joint development of the two abovementioned organizations which is described in this work. On the generated recombinant influenza viruses and a method of their application are submitted at the national patent applications (entitled: «The recombinant strains of influenza A virus expressing brucellosis immunodominant proteins destined for specific prophylaxis of brucellosis», registration number is 2013/7090.1). At the moment we are preparing an application for an international patent. In adherence to the Virology Journal guidelines, RIBSP and HSC Development GmbH will make freely available any materials and information described in the publication that are reasonably requested by others for the purpose of academic, non-commercial research. This does not alter the authors’ adherence to all the Virology Journal policies on sharing data and materials.

## Authors’ contributions

KT, AS and ZhK conceived and designed the experiments. ZhK, KS, BY, ShR, NZ, NA, NS, BKh, IK, BF and AE performed the experiments. KT, AS and AE analyzed the data. ShR, NZ, NA and NS contributed to the work with data analysis and interpretation of results. KT wrote the paper. All authors read and approved the final manuscript.
